# Do Interventions to Prevent Lifestyle-Related Diseases Reduce Healthcare Expenditures? A Randomized Controlled Clinical Trial

**DOI:** 10.2188/jea.JE20100095

**Published:** 2011-01-05

**Authors:** Akira Babazono, Kazuaki Kuwabara, Akihito Hagiihara, Jun Nagano, Reiko Ishihara

**Affiliations:** 1 Department of Health Care Administration and Management, Graduate School of Medical Sciences, Kyushu University, Fukuoka, Japan; 2 Institute of Health Science, Kyushu University, Fukuoka, Japan; 3 Department of Healthcare Management, College of Healthcare Management, Miyama, Fukuoka, Japan

**Keywords:** health policy, randomized controlled trial, prevention, lifestyle-related diseases, health care expenditures

## Abstract

**Background:**

In 2008, the Japanese government implemented a program of health lifestyle interventions to reduce health care expenditure. This study evaluated whether these interventions decreased health care expenditures.

**Methods:**

The study enrolled 99 participants insured by Japanese National Health Insurance, who, in our previous study conducted in 2004, were allocated by random sampling into an intervention group (50 participants) and a control group (49 participants). In the intervention group, we used a health support method that facilitated the attainment of goals established by each participant. The control group received instruction in exercise, as well as health support using publically available media. Although 3 participants in the intervention group and 9 participants in the control group did not participate in a follow-up health examination 1 year after the intervention, the health care expenditures of all initial participants were assessed. Expenditures before and after the intervention were compared within and between groups. Data on health care expenditures were obtained from inpatient, outpatient, pharmacy, and dental health insurance claims.

**Results:**

After the intervention, the pharmacy and dental expenditures were significantly higher in the intervention group, while the pharmacy expenditure was significantly higher in the control group. However, there was no significant difference in any medical expenditure item between the intervention and control groups before or after the intervention.

**Conclusions:**

No significant differences were observed in short-term medical expenses for any medical expenditure item after a lifestyle intervention.

## INTRODUCTION

The increase in health care expenditures is a problem common to all developed countries. Attempted control measures have included regulation of health resources made available by national policies, increased fees in the form of consumer copayments, and the manipulation of insurance payouts; however, the deficiency in the quality of medical services often persists. Therefore, attempts have been made to control medical expenditure through disease prevention and control.^1^^,^^2^

Lifestyle-related diseases accounted for about 60% of deaths in Japan in 2007,^3^ and health care expenditure for these diseases is believed to have accounted for a high percentage of the country’s total health care expenditures (33 trillion yen) in that year.^3^ As part of Japan’s health care reform plan (2008–2012), insurers are now required to provide certain health examinations and guidance related to metabolic syndrome.^3^ To reduce the number of persons with or at high risk for lifestyle-related diseases such as diabetes mellitus, the reform plan requires insurers to determine the risk for metabolic syndrome, based on the patient’s level of obesity (as determined by using abdominal circumference and BMI), blood glucose and lipid levels, blood pressure, and the presence or absence of a smoking habit.^4^ Then, insurers must offer health guidance interventions according to the degree of risk in insured adults aged 40 years or older. Financial penalties can be imposed on insurers that fail to adhere to established benchmarks, such as rates of participation in health examinations and guidance sessions.^5^

There have been many economic evaluations of disease prevention programs worldwide.^6^^–^^12^ In Japan, ecological studies demonstrated that participation rates in health examinations were negatively correlated with health care expenditures.^13^^,^^14^ In addition, several studies evaluated the effects of interventions that aim to prevent lifestyle-related disease on health care expenditures.^15^^,^^16^ However, there have been few randomized controlled studies of the effects of lifestyle interventions on a country’s health care expenditure. We maintain that it is important to use randomized controlled trials to gather such evidence because insurers are subject to financial penalties if they fail to meet the targeted indicators.

## METHODS

As part of a randomized controlled study in 2004, we conducted a lifestyle intervention for adults insured by the Japanese National Health Insurance system. We observed increases in participants’ mean number of pedometer steps and intake of yellow/green vegetables up to 1 year after the intervention^17^; the study design and findings are described below. Participation in the intervention was recommended to people who did not receive regular medical care in a medical institution but required health guidance for hypertension (defined as a systolic blood pressure of 130 to 159 mm Hg or diastolic pressure of 85 to 99 mm Hg) or diabetes mellitus (defined as a glycosylated hemoglobin [HbA1c] level of 5.6% or higher), based on the results of health examinations performed in 2004.

A total of 99 participants (42 men and 57 women) were randomly allocated to an intervention group (50 participants) or a control group (49 participants). In the intervention group, we introduced a new health support method in which the internal motivation of participants was assessed, after which they attempted to modify their behavior by attaining goals they themselves had established. The control group received an explanation of the results of their health examination, exercise instruction, and support using publicly available media, such as brochures, which are the most common source of patient education in community health projects. Health examination data obtained at the start of the intervention were compared with those of a health examination conducted 1 year later in both the intervention and control groups. Three participants in the intervention group and 9 in the control group did not participate in the post-intervention health examination. Thus, the health outcome analyses included 47 participants in the intervention group and 41 participants in the control group. The numbers of pedometer steps per day at baseline and at 1 year in the intervention group were 7345 and 10 373, respectively, a significant increase. In the control group, the corresponding values were 7196 and 6815 steps, a nonsignificant change. The difference between the pre- and post-intervention values was significantly larger in the intervention group. The numbers of participants in the intervention group who ate green/yellow vegetables once a day or less and twice a day or more were 30 (65.2%) and 16 (34.8%), respectively, at baseline and 23 (50.0%) and 23 (50.0%) after 1 year. In the control group, the corresponding values were 29 (70.7%) and 12 (29.3%) at baseline and 29 (70.7%) and 12 (29.3%) after 1 year. The intake distribution of the 2 categories of green/yellow vegetables 1 year after the intervention significantly differed between the intervention and control groups. However, there was no significant difference between the intervention and control group in body weight, body mass index, systolic blood pressure, diastolic blood pressure, levels of total cholesterol, triglyceride, high-density lipoprotein (HDL) cholesterol, low-density lipoprotein (LDL) cholesterol, or HbA1c, or results on the General Health Questionnaire 30 (GHQ30).

Information on inpatient, outpatient, pharmacy, and dental expenditures was obtained from health insurance claims for 2002 (1 April 2002 to 31 March 31 2003), 2003 (1 April 2003 to 31 March 2004), 2005 (1 April 2005 to 31 March 2006), and 2006 (1 April 2006 to 31 March 2007). The pre-intervention values were defined as the mean values for number of claims or serviced days and health care expenditures per year for inpatient, outpatient, and dental care in 2002 and 2003; post-intervention values were defined as the values from 2005 and 2006. Normal, logarithm transformation was conducted as follows:

Log(x+1); x=health care indicator

The unpaired *t*-test was used to compare log-transformed indicators between the intervention and control groups before and after the intervention. In addition, pre- and post-intervention values were compared within each group by using the paired *t*-test. The health care expenditures for all initial participants were assessed.

All participants gave informed consent for use of the data, and ethical approval was obtained from the Ethics Committee of the Institute of Health Science at Kyushu University. All analyses were performed using the statistical package SPSS (version 15.0), and the exchange rate used was 110 yen to 1 US dollar, which was the approximate average rate in 2004.

## RESULTS

Table 1 shows the characteristics of the participants in the intervention and control groups. The intervention group (50 participants) included 21 men (42.0%) and 29 women (58.0%), while the control group (49 participants) included 21 men (42.9%) and 28 women (57.1%). The mean age was 64.3 years (65.2 years in men and 63.6 years in women) in the intervention group and 64.5 years (63.4 years in men and 65.3 years in women) in the control group. No statistical difference was observed between the intervention and control groups. In both groups, the most common lifestyle-related disease was hypertension, followed by diabetes mellitus. In the intervention group, 21 participants (42.0%) had hypertension, 16 (32.0%) had diabetes mellitus, 6 (12.0%) had both diseases, and 19 (38.0%) had neither; in the control group 24 participants (49.0%) had hypertension, 16 (32.7%) had diabetes mellitus, 9 (18.4%) had both diseases, and 18 (36.7%) had neither. No significant difference in disease distribution was observed using the chi-square test. All participants were alive and thus available for follow-up after the study period.

**Table 1.** Age, sex, and disease distributions of cohort after randomization

Characteristic	Intervention group	Control group
Mean age, y (SD)	64.3 (7.1)	64.5 (7.9)

Sex, no. (%)		
Male	21 (42.0)	21 (42.9)
Female	29 (58.0)	28 (51.1)
Disease status, no. (%)		
Hypertension^a^	21 (42.0)	24 (49.0)
Diabetes mellitus^b^	16 (32.0)	16 (32.7)
Both	6 (12.0)	9 (18.4)
Neither	19 (38.0)	18 (36.7)
Total	50 (100.0)	49 (100.0)

Abbreviation: SD, standard deviation.

^a^Defined as a systolic blood pressure of 130–159 mm Hg or a diastolic pressure of 85–99 mm Hg.

^b^Defined as glycated hemoglobin (HbA1c) ≥5.6%.

Table 2 shows the mean numbers of inpatient, outpatient, and dental claims per year before and after the intervention. Before the intervention, the mean number of inpatient claims was 0.1 in both the intervention group and control group; after the intervention, the respective numbers were 0.1 and 0.2. The mean number of outpatient claims was 6.3 in the intervention group and 5.1 in the control group before the intervention and 10.4 and 8.7, respectively, after the intervention. The mean number of pharmacy claims was 0.9 in the intervention group and 0.8 in the control group before the intervention and 4.8 and 5.3, respectively, after the intervention. The mean number of dental claims was 1.4 in the intervention group and 1.3 in the control group before the intervention and 2.8 and 1.8, respectively, after the intervention. No significant differences were observed before or after the intervention in the mean log-transformed numbers of inpatient, outpatient, or dental claims between the 2 groups. However, after the intervention, the mean log-transformed numbers of outpatient, pharmacy, and dental claims were significantly higher in the intervention group, while the mean log-transformed numbers of outpatient and pharmacy claims were significantly higher in the control group.

**Table 2.** Mean numbers of inpatient, outpatient, pharmacy, and dental claims per year

	Baseline		After intervention		Difference	
			
	Intervention group	Control group	Intervention group	Control group	Intervention group	Control group
	Mean (SD)	Mean (SD)	Mean (SD)	Mean (SD)	Mean (SD)	Mean (SD)
Inpatient	0.1 (0.3)	0.1 (0.2)	0.1 (0.3)	0.2 (0.7)	0.0 (0.4)	0.2 (0.8)
Log-transformed value	0.0 (0.1)	0.0 (0.1)	0.0 (0.1)	0.0 (0.1)	0.0 (0.0)	0.0 (0.1)

Outpatient	6.3 (6.3)	5.1 (5.5)	10.4 (8.3)	8.7 (7.8)	4.2 (5.9)	3.6 (5.7)
Log-transformed value	0.7 (0.4)	0.6 (0.4)	0.9 (0.4)	0.8 (0.4)	0.2^b^ (0.3)	0.2^a^ (0.4)

Pharmacy	0.9 (1.9)	0.8 (2.1)	4.8 (5.4)	5.3 (6.3)	3.9 (4.8)	4.5 (6.5)
Log-transformed value	0.2 (0.3)	0.1 (0.3)	0.6 (0.4)	0.6 (0.4)	0.4^b^ (0.4)	0.5^b^ (0.5)

Dental	1.4 (1.8)	1.3 (1.6)	2.8 (3.4)	1.8 (2.2)	1.5 (3.7)	0.5 (2.4)
Log-transformed value	0.3 (0.3)	0.3 (0.3)	0.5 (0.3)	0.3 (0.3)	0.2^b^ (0.4)	0.1 (0.4)

Abbreviation: SD, standard deviation.

^a^*P* < 0.01, ^b^*P* < 0.001.

Table 3 shows the mean numbers of inpatient, outpatient, and dental serviced days of care per year. The mean number of inpatient serviced days was 1.3 in the intervention group and 0.5 in the control group before the intervention and 1.6 and 1.9, respectively, after the intervention. The mean number of outpatient serviced days was 16.7 in the intervention group and 12.8 in the control group before the intervention and 20.6 and 18.1, respectively, after the intervention. The mean number of dental serviced days was 5.2 in the intervention group and 4.5 in the control group before the intervention and 8.3 and 7.2, respectively, after the intervention. No significant differences were observed in the mean log-transformed number of any category of serviced days between the 2 groups before or after the intervention. However, after the intervention, the mean log-transformed numbers of outpatient and dental serviced days were significantly higher in the intervention group, while only the mean log-transformed number of outpatient serviced days was significantly higher in the control group.

**Table 3.** Mean numbers of inpatient, outpatient, and dental serviced days per year

	Baseline		After intervention		Difference	
			
	Intervention group	Control group	Intervention group	Control group	Intervention group	Control group
	Mean (SD)	Mean (SD)	Mean (SD)	Mean (SD)	Mean (SD)	Mean (SD)
Inpatient	1.3 (5.2)	0.5 (2.4)	1.6 (4.2)	1.9 (7.7)	0.3 (6.7)	1.4 (8.2)
Log-transformed value	0.1 (0.3)	0.1 (0.2)	0.2 (0.4)	0.1 (0.3)	0.0 (0.5)	0.1 (0.4)

Outpatient	16.7 (26.3)	12.8 (20.9)	20.6 (23.1)	18.1 (19.4)	3.9 (18.3)	5.3 (21.7)
Log-transformed value	0.9 (0.6)	0.8 (0.5)	1.1 (0.5)	1.0 (0.6)	0.2^a^ (0.4)	0.2^a^ (0.5)

Dental	5.2 (7.5)	4.5 (7.4)	8.3 (9.1)	7.2 (11.4)	3.1 (10.0)	2.7 (10.2)
Log-transformed value	0.5 (0.5)	0.5 (0.5)	0.8 (0.5)	0.6 (0.5)	0.3^a^ (0.6)	0.2 (0.6)

Abbreviation: SD, standard deviation.

^a^*P* < 0.01.

Table 4 shows mean inpatient, outpatient, pharmacy, dental, and total expenditures per year. The mean inpatient expenditure was 21 241 yen (US$193) in the intervention group and 18 304 yen ($166) in the control group before the intervention and 47 832 yen ($435) and 86 366 yen ($785), respectively, after the intervention. The mean outpatient expenditure was 81 001 yen ($736) in the intervention group and 57 118 yen ($519) in the control group before the intervention and 135 176 yen ($1229) and 92 600 yen ($842), respectively, after the intervention. The mean pharmacy expenditure was 5209 yen ($47) in the intervention group and 4446 yen ($40) in the control group before the intervention and 30 209 yen ($275) and 27 560 yen ($251), respectively, after the intervention. The mean dental expenditure was 24 010 yen ($218) in the intervention group and 27 204 yen ($247) in the control group before the intervention and 41 730 yen ($379) and 34 687 yen ($315), respectively, after the intervention. The mean total expenditure was 131 460 yen ($1195) in the intervention group and 107 073 yen ($973) in the control group before the intervention and 254 948 yen ($2318) and 241 212 yen ($2193), respectively, after the intervention. No significant differences were observed in any log-transformed expenditure item between the intervention and control groups before or after the intervention. However, mean log-transformed pharmacy and dental expenditures were significantly higher after the intervention in the intervention group, while only log-transformed pharmacy expenditure was significantly higher after the intervention in the control group. There was no significant increase in log-transformed total expenditure in either group.

**Table 4.** Mean inpatient, outpatient, pharmacy, dental, and total expenditures per year

		Baseline				After intervention				Difference			
				
		Intervention group		Control group		Intervention group		Control group		Intervention group		Control group	
		Mean (SD)		Mean (SD)		Mean (SD)		Mean (SD)		Mean (SD)		Mean (SD)	
Inpatient	Yen	21 241	(83 353)	18 304	(102 796)	47 832	(150 736)	86 366	(317 051)	26 592	(161 275)	68 062	(338 107)
	US$	193	(758)	166	(935)	435	(1370)	785	(2882)	242	(1466)	619	(3074)
Log-transformed value		0.67	(1.70)	0.32	(1.28)	0.76	(1.91)	0.79	(1.96)	0.09	(2.30)	0.47	(2.45)

Outpatient	Yen	81 001	(177 824)	57 118	(91 082)	135 176	(202 087)	92 600	(10 087)	54 175	(93 985)	35 482	(95 756)
	US$	736	(1617)	519	(828)	1229	(1837)	842	(92)	493	(854)	323	(871)
Log-transformed value		3.99	(1.71)	3.97	(1.58)	4.43	(1.57)	4.27	(1.56)	0.45	(1.70)	0.29	(1.61)

Pharmacy	Yen	5209	(16 600)	4446	(13 941)	30 209	(52 823)	27 560	(38 243)	25 000	(49 352)	23 114	39 471
	US$	47	(151)	40	(127)	275	(480)	251	(348)	227	(449)	210	359
Log-transformed value		1.22	(1.82)	1.01	(1.73)	3.43	(1.71)	3.41	(1.73)	2.22^a^	(1.93)	2.40^a^	(2.37)

Dental	Yen	24 010	(34 070)	27 204	(42 444)	41 730	(53 508)	34 687	(42 765)	17 721	(61 278)	7482	(56 548)
	US$	218	(310)	247	(386)	379	(486)	315	(389)	161	(557)	68	(514)
Log-transformed value		2.44	(2.29)	2.46	(2.28)	3.73	(1.80)	3.13	(2.13)	1.29^a^	(2.62)	0.67	(2.51)

Total	Yen	131 460	(249 451)	107 073	(194 980)	254 948	(323 927)	241 212	(394 552)	123 488	(217 233)	134 140	(413 581)
	US$	1195	(2268)	973	(1773)	2318	(2945)	2193	(3587)	1123	(1975)	1219	(3760)
Log-transformed value		4.48	(1.42)	4.54	(1.10)	4.90	(1.33)	4.70	(1.51)	0.42	(1.54)	0.16	(1.43)

Abbreviation: SD, standard deviation.

^a^*P* < 0.001.

## DISCUSSION

Many health projects aim to improve the health of the insured while reducing national health care expenditures and maintaining financial balance. Because of increases in health care expenditures due to aging populations, as well as decreases in insurance premiums due to the global economic downturn, many insurers have had financial difficulties, and more effective health promotion projects have been advocated. The Japanese government aims to reduce the number of lifestyle-related diseases by 25%, with the expectation of a subsequent reduction in health care expenditures.^5^ Thus, the results of the present randomized controlled study, in which health care expenditures of an intervention group that changed health behaviors were compared with those of a control group that did not make such changes, would be expected to have policy implications.

The intervention program in this study was based on a lifestyle support model that considered the participants’ mental health level, facilitated the development of appropriately personalized goals, and promoted independence by encouraging self-efficacy. An evaluation conducted 1 year after the lifestyle intervention showed significant increases in intake of green/yellow vegetables and exercise (mean number of pedometer steps per day) in the intervention and control groups, suggesting favorable long-term influences on health outcome. However, no difference in these items was observed between the intervention and control groups before or after the lifestyle intervention. After the lifestyle intervention, the numbers of outpatient, pharmacy, and dental claims, the numbers of outpatient and dental serviced days, and pharmacy and dental expenditures were significantly higher in the intervention group, while the numbers of outpatient and pharmacy claims, numbers of outpatient and pharmacy serviced days, and pharmacy expenditure per year were significantly higher in the control group. These results are consistent with a meta-analysis of data from 2947 people who participated in an individualized health promotion program in Japan.^18^ In that study, similar, significant increases were seen after a lifestyle intervention.

Why did the numbers of outpatient and serviced days, the number of pharmacy claims, and pharmacy expenditure increase in both groups after the intervention? First, participants were 3 years older at the end of the intervention. Therefore, increases in the numbers of outpatient claims and serviced days and the number of pharmacy claims and pharmacy expenditures per year would be expected to increase with advancing age. In addition, at the time of the health examination they underwent as part of the intervention, some participants received the information that they had a high risk of certain lifestyle-related diseases. Thus, it is possible that they were motivated to consult with physicians because of anxiety regarding such disease risks^19^^,^^20^ or a desire to seek further health assessment or professional advice. Moreover, it is possible that through the intervention, people with poor dental health in the intervention group were advised to visit dental clinics, because many dental conditions are related to lifestyle-related diseases.^21^^,^^22^ By encouraging people to visit physicians, health examinations and guidance might increase outpatient visits and pharmacy expenditure. Therefore, the absence of differences in health care expenditures between the 2 groups indicates that our lifestyle intervention did not reduce health care expenditures in the short term.

There were some limitations in this study. First, the sample size was small. Thus, a large-scale randomized controlled study is necessary. Second, because there was no disease-specific analysis, health care expenditures not associated with lifestyle-related diseases would be included in the results, which could lead to misclassification bias. However, there was no inpatient claim for which expenditure exceeded 2 million yen ($18 182). Therefore, it is unlikely that inpatient claims substantially changed the mean value of the medical indicator. Third, more than 1 outpatient claim might have been submitted if a patient visited more than 1 specialty during the study period. In such cases, the number of outpatient claims or serviced days might be inflated. Finally, the period of analysis included only the 2 years after the intervention. To fully evaluate effects on health care expenditures for lifestyle-related diseases, a longer follow-up period is necessary. In addition, it is necessary to integrate the results of economic evaluations from small-scale intervention studies conducted in numerous local areas.

Regarding the “specific health examination and instruction projects” described in the Elderly Health Care Security Act,^23^ which was introduced in 2008 as a mandate to insurers, the results of such projects must be assessed in terms of structure, process, and results. Only studies of the structure and frequency of project implementation have been performed since 2008, as these health projects are still at an early stage. The most important measure in preventing lifestyle-related diseases is the improvement of health habits. People must voluntarily change their behavior and maintain this improved behavior, thereby forming new lifestyle habits. However, it is difficult to improve lifestyle habits that have formed over a long period of time and to maintain such improvements. Policymakers need to develop appropriate disease management programs to ensure efficient use of health care resources. In designing health projects that use public funds, studies such as the present analysis are necessary to determine whether program choices lead to better cost performance and if specific interventions for behavior change require monitoring or refinement.
